# How COVID-19 pandemic changed our communication with families: losing nonverbal cues

**DOI:** 10.1186/s13054-020-03035-w

**Published:** 2020-06-05

**Authors:** Annachiara Marra, Pasquale Buonanno, Maria Vargas, Carmine Iacovazzo, Eugene Wesley Ely, Giuseppe Servillo

**Affiliations:** 1 Department of Neurosciences, Reproductive and Odontostomatological Sciences, University of Naples, Federico II, Via Pansini 5, 80131 Naples, Italy; 2 Critical Illness, Brain Dysfunction, and Survivorship (CIBS) Center, Vanderbilt University School of Medicine, Nashville, TN USA; 3 Department of Medicine, Division of Allergy, Pulmonary and Critical Care Medicine, Vanderbilt University School of Medicine, Nashville, TN USA; 4 Geriatric Research Education and Clinical Center (GRECC), Department of Veterans Affairs Medical Center, Tennessee Valley Healthcare System, Nashville, TN USA; 5 Center for Health Services Research, Vanderbilt University School of Medicine, Nashville, TN USA

The outbreak of coronavirus disease 2019 (COVID-19) has created a global health crisis that has had a deep impact on the way we communicate with patients and their relatives in all the COVID-19 care settings, given the need to maintain isolation and social distancing [1]. To be highly effective, communication in medical encounters must capitalize on both verbal and nonverbal aspects. Both of these have been highly compromised in the COVID-19 experiences both in hotbed sites and in affected but more controlled settings. Loved ones of COVID-19 patients are suffering in unique ways as a result of adaptions in our communication.

Dealing with emotion is as important as relaying information about diagnosis and prognosis, detecting and recognizing emotions as legitimate enables you to create trust and establish therapeutic alliance [2]. Nonverbal communication is established by eye contact, posture, tone of voice, head nods, gesture, and the postural position. Empathy is of great significance for better healthcare outcomes as part of a warm and friendly communication style. Communication between intensive care unit (ICU) staff and patients’ families is essential in critical care medicine: relatives rate *communication* skills as just as valuable as clinical skills, or even more so (Fig. 1).

**Fig. 1:**
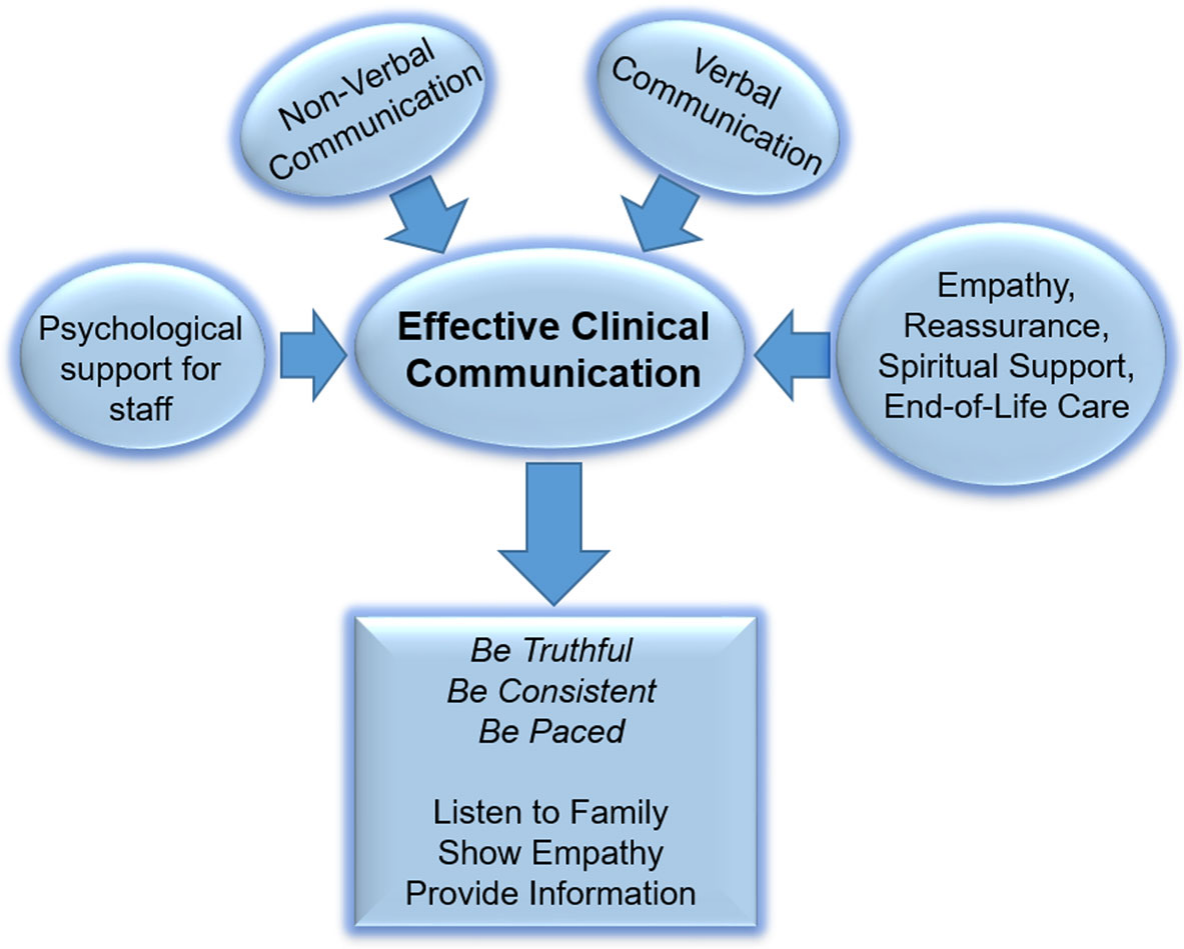
Effective clinical communication with family in ICU

The relatives of critically ill patients are at increased risk for traumatic stress symptoms (PTSS, 57%), anxiety (80%), and depression (70%) [3]. These highlighted nonverbal aspects of our communication are thwarted, ineffective, and impaired during the COVID-19 pandemic. During this dramatic scenario, clinicians are being confronted with new communication tasks that we have not faced before, generating extra measures of apprehension, uncertainty, and fear [4].

Now we have to acknowledge the fear, sadness, and anxiety that patients’ families experience as they are isolated from the ones they love in life, often as they are dying. We have to do this without looking at the families in the eyes, without the possibility of providing comfort through an embrace, the touch of a hand, or love through crying with them. Our information is given by telephone, video call, or e-mail, and physicians have the challenging aim of compensating for these egregious communication gaps through other nonverbal tools such as the tone of our voice, pause, and inflection. The importance of communication during this health emergency is witnessed by the increasing of publication of national and international guidelines. Multiple Italian societies (SIAARTI, Aniarti, SICP, SIMEU) released a joint document on “How to communicate with families living in complete isolation.” Communication must be unequivocal, truthful, reasoned, and appropriate to the recipient’s ability to understand their emotional state and life situation, with particular attention to frailty, suggesting hope by not creating or encouraging unrealistic expectations, reconstruct the patient’s preferences and values so as to respect their autonomy [5]. Patients and their families perceive not only the clinical results but also the personal attitudes, closeness, and psychological support from the care teams [6]. This perception of genuine participation by the health worker in the course of the treatment is especially important when a patient dies and may influence the whole process of grief.

The heavy workloads and emotional stress that this emergency is causing to health workers may compromise the health worker’s ability to act effectively and efficiently [7]. Health workers’ mental and emotional balance must be taken into consideration and protected alongside that of the family. Both must be viewed as a priority of pandemic after-care in the recovery process to come for us as a healing society.

## Data Availability

Not applicable
